# Visualizations of problem-posing activity sequences toward modeling the thinking process

**DOI:** 10.1186/s41039-016-0042-4

**Published:** 2016-08-17

**Authors:** Ahmad Afif Supianto, Yusuke Hayashi, Tsukasa Hirashima

**Affiliations:** 1 Department of Information Engineering, Graduate School of Engineering, Hiroshima University, 1-4-1 Kagamiyama, Higashi-hiroshima, Hiroshima, 739-8527 Japan; 2 Department of Informatics, Faculty of Computer Science (FILKOM), Brawijaya University, 8 Veteran Road, Malang, 65145 Indonesia

**Keywords:** Problem-posing process, Problem state space, Visualizations

## Abstract

Problem-posing is well known as an effective activity to learn problem-solving methods. Although the activity is considered in contributing to the understanding of the problem’s structure, it is not clear how learners could understand it through the activity. This study proposes a method to offer a visual representation for analyzing the problem-posing activity sequence in MONSAKUN, a digital learning environment for problem-posing of arithmetic word problems via sentence integration. This system requires users to pose a problem through combinations of given simple sentences based on the requirement. The system writes every single action into logs as sequences of problem-posing activity. The sequences are considered to represent the thinking processes of learners. The thinking process reflects their understanding and misunderstanding about the structure of the problems. This study created visualizations of learners’ problem-posing processes from the data obtained through the practical use of MONSAKUN, including the states in which many learners had difficulties finding the correct answer. In this study, we refer to such states as “trap states.” In MONSAKUN, a trap state is a combination of simple sentences where many learners tend to make and need relatively more actions to obtain the correct answer. As the result of the visualization and analysis of the data, some trap states have been identified, and they changed for each trial in the same problem.

## Introduction

### The importance of problem-posing activity in mathematics

Problem-posing is considered to be an essential part of mathematical activity (Brown and Walter 1993). Problem-posing involves generating new problems and questions aimed at exploring a given situation as well as reformulating a problem during the course of solving a related problem (Silver 1994). Providing students with an opportunity to pose their own problems can foster flexible thinking, enhance problem-solving skills, broaden their perception of mathematics, and enrich and consolidate basic concepts (Brown and Walter 1993; English 1996).

The development of problem-posing skills for learners is one of the principal aims of mathematics learning, and it should occupy a central role in mathematics activities (Crespo 2003). Moreover, problem-posing activities could provide us with valuable insights into children’s understanding of mathematical concepts and processes, as well as their perceptions of, and attitudes toward, problem-solving and mathematics in general (Brown and Walter 1993). For the improvement of students’ learning in problem-posing, it is important to develop an understanding of the developmental status of learners’ thinking and reasoning. The more information that can be obtained about what learners know and how they think, the more opportunities could be created for the enhancement of learners’ success (Cai 2003).

Learning by problem-posing in conventional classrooms has been studied, and several investigations have confirmed that problem-posing is a promising learning activity. English (1998) investigated the problem-posing abilities of children who displayed different profiles of achievement in number sense and novel problem-solving. Silver and Cai (1996) found that students generated a large number of solvable mathematical problems, many of which were syntactically and semantically complex, and that nearly half the students generated sets of related problems.

### Assessment of the posed problems

In problem-posing, assessment of each posed problem and assistance based on it are necessary (Hirashima et al. 2007). Teacher assessment of posed problems encompasses learners’ development of diverse mathematical thinking (English 1997). The other one is investigating learners’ behavior of the problem-posing process by extending their posed problems (Singer et al. 2011). However, since learners are usually allowed to pose several kinds of problems, including a large range of them, it can be challenging for teachers to complete assessment and feedback for the posed problems in the classroom.

Technology-enhanced approaches have been used to realize learning by problem-posing, especially in regard to assessment and feedback. A networked, question-posing learning system enabling students to pose questions was developed by Yu et al. (2003–2004). A novel way for merging assessment and knowledge sharing using an online Question-Posing Assignment (QPA) has been examined (Barak and Rafaeli 2004). An online learning system with a focus on student-question generation strategy, called QuARKS (Question-Authoring and Reasoning Knowledge System) (Yu 2009), was adopted and the effects of student question-generation on civics and citizenship (Yu and Pan 2014) and English learning (Yu et al. 2015) have been reported. Student academic achievement, question-generation performance, learning satisfaction and learning anxiety, as well as learning motivation have been investigated. Self- and peer-assessed posed problems were examined in these studies.

In contrast, diagnosis functions that can assess and give automatic feedback to each posed problem have been proposed (Nakano et al. 1999; Hirashima et al. 2000). This automatic way of diagnosis-facility assessment is called agent assessment. Furthermore, learning environment systems that use practical agent assessment have been developed (Hirashima et al. 2008a, 2008b, 2014). This research aimed at the practical realization of agent assessment in order to understand the process of learners’ problem-posing, so that it could be analyzed. The current study uses the same kind of learning environment system that the above studies in agent assessment used.

A new design of the problem-posing learning environment using agent assessment is sentence integration (Hirashima et al. 2007), named MONSAKUN. In problem-posing via sentence integration, several simple sentences are provided to a learner. The learner, then, selects the necessary sentences and arranges them in an appropriate order. This approach makes simple and goal-oriented problem-posing tasks possible even for lower elementary students, while maintaining its value as a viable learning method and practical approach to data collection in an interactive learning environment. The other approaches are called Questions Generation (Silver and Cai 1996) and Animal Watch (Arroyo and Woolf 2003). The problem-posing task in the Questions Generation approach asked students to pose three questions that could be answered on the basis of some given information. An investigation was conducted to find whether responses were classified as mathematical questions, non-mathematical questions, or statements. The Animal Watch is an Intelligent Tutoring System (ITS) that teaches mathematics with word problems, which are integrated into narratives about endangered species to engage student interest and help them appreciate the value of learning mathematics. Students authored one episode for their adventure and called it something along the lines of “Meet the (endangered species).” Students then authored addition, subtraction, and multiplication word problems related to the number facts that had collected for their species. Teacher supervision of the resultant word problems was then held in order to clarify students’ misconceptions.

The use of the sentence-integration method was proven to improve learning by problem-posing for students in lower elementary school. A long-term evaluation of the system was carried out, and the study confirmed that it was interesting and useful for learning (Hirashima et al. 2008a). Moreover, the system also improved the problem-solving ability of low-performance students (Hirashima et al. 2008b). In 2011, a task model of problem-posing was developed that dealt with not only the forward-thinking problem but also the reverse-thinking problem (Hirashima and Kurayama 2011). Yamamoto et al. (2013) reported on learning environments that utilized online connected media tablets and their practical uses among first-grade students. The results showed that the practice of posing problems improved learners’ abilities not only in problem-posing but also in problem-solving. Finally, an interactive learning environment for learning by problem-posing based on the “triplet structure model” was developed and practically used (Hirashima et al. 2014). In practical use, it was confirmed that learning by problem-posing with MONSAKUN was a useful learning method.

### Analysis of learning activity using MONSAKUN

Several studies have specifically addressed the analysis of learning activities using MONSAKUN. Hirashima et al. (2007) analyzed whether learners could pose problems based on the logs of the system, which shows and discusses the number of posed problems and correct problems. Hirashima et al. (2008a) and Hirashima and Kurayama (2011) analyzed the learning effects of MONSAKUN by comparing pre-test and post-test problem-solving and posing scores. Hasanah et al. (2015a) conducted further analysis on this topic. Their study examined learners’ thinking processes based on the first selected sentence in assignments on MONSAKUN. Binomial tests on students’ first card selections in each assignment were implemented to analyze the results and found that the selection changed based on different types of approaches, types of stories, and students’ previous experiences with the exercises. Even though many studies have analyzed the log data from MONSAKUN, few studies have used visualization to exploit the potential of learning activities in that environment.

Visualization of log data is a useful approach to interpreting learners’ thinking processes. The purpose of visualization is to “amplify cognition” regarding data (Card et al. 1999). Visualization could be fully leveraged to better understand via step-by-step data logs generated by learning environments. Anscombe (1973) suggested that a computer should use both calculations and graphs; both sorts of output should be studied because each of them would contribute to understanding. Visualization could help to avoid misinterpretation of data. Shneiderman (2002) claimed that integration of both data mining and information visualization to invent discovery tools could enable more effective exploration and promote responsibility.

There has been considerable work exploring the importance of visualization to externalize the activity of learners. Some of them have conducted design and visualization learning processes in a computer-supported collaborative learning environment (Janssen et al. 2007a; Tan et al. 2008), visualized and externalized the activity of groups working together in collaborative learning participation (Janssen, Erkens, Kanselaar, & Jaspers, 2007b; Rabbany et al. 2011), and visualized the learning interaction with respect to the collaborative and learning attitudes of each participant (Hayashi et al. 2013). On individual learning, systems that collect detailed, real-time data on learner behavior and interpret those data by drawing on behavioral research have been developed (Macfadyen and Sorenson 2010). Anjewierden et al. (2011) developed the adaptive learning environment. This system could monitor learner behavior through the actions learners perform and identify patterns that point to systematic behavior using visual representation. Moreover, Johnson et al. (2011) developed the visualization that uses a tree structure to provide an overview of class performance for easy navigation and exploration of student behavior.

### Motivation and research purpose

In general problem-posing exercises where learners pose problems freely, it is difficult for students to pose problems and for teachers to analyze the problems posed by students. The learning environment for problem-posing exercises, MONSAKUN, resolves this difficulty with the problem-posing method, with problem-posing by sentence integration. In this method, learners pose problems meeting certain requirements by combining three simple sentences from the given sentences. By using this method, the opportunities for learners to pose problems increase, feedback to learners according to their mistakes is provided, and for the teacher, checking the validity of posed problems becomes easier.

From the results of previous studies with MONSAKUN, lessons and exercises with it improve not only learners’ problem-posing but also their problem-solving (Yamamoto et al. 2012). In addition, from the preliminary analysis of sentence selection in problem-posing, learners change their approach to problem-posing after they have experienced posing the same type of story (Hasanah et al. 2015a). Although posing problems in the learning environment is considered to contribute to the understanding of the problem’s structure, it is not clear how learners could understand it through the activity. Therefore, it is essential to conduct discovery learning and to generate inferences of learners’ thinking from their behavior in learning environments. Discovery learning plays a role in increasing learners’ motivation, while creating more opportunities for learners to assess how well they could overcome obstacles, which may improve learning (Reiser et al. 1998).

The learning environment used in this study is MONSAKUN. MONSAKUN records the posed problems created by learners step-by-step as log data. The purpose of this study is to offer a visual representation for analyzing the problem-posing processes of learners in MONSAKUN. These visualizations could detect important circumstances according to the changes in their thinking processes. By this detection, we would be able to provide information regarding a situation in which many learners experience bottlenecks and misunderstanding of the structure of the problems. The people who benefit from the results of this approach are teachers and researchers. If teachers understand the difficulties faced by learners, they can provide more helpful feedback. If researchers understand the difficulties faced by learners, they can consider and develop functions to overcome them. In the current state of this study, we propose a method to visualize learner’s actions from the log data of MONSAKUN, to extract some information from it, and to analyze the results.

The remainder of this paper is organized as follows. The next section describes how the MONSAKUN problem-posing learning environment works based on the triplet structure model. The next section details the procedure of visualization of the problem-posing process proposed in this study. The next section applies the procedure of visualization to a case study on the practical use of MONSAKUN followed by discusses learners’ behavior from the visualization of data collected in the case study. Finally, we conclude by summarizing the results and offer suggestions for future study in the last section.

### The problem-posing learning environment based on the triplet structure model

This section discusses how the MONSAKUN problem-posing learning environment works based on the triplet structure model. First, we describe the environment of MONSAKUN and its components and their functions. Then, we explain how the activity of learners works in MONSAKUN so that it can be exploited as useful information. Second, the underlying model of MONSAKUN, called the triplet structure model, is explained. Based on the consideration of problem structure in the triplet structure model, the task model of problem-posing as sentenceintegration is developed. Finally, the constraints to form arithmetic word problems based on the task model are discussed and followed by a number of case examples.

### MONSAKUN as a problem-posing learning environment

**Fig. 1:**
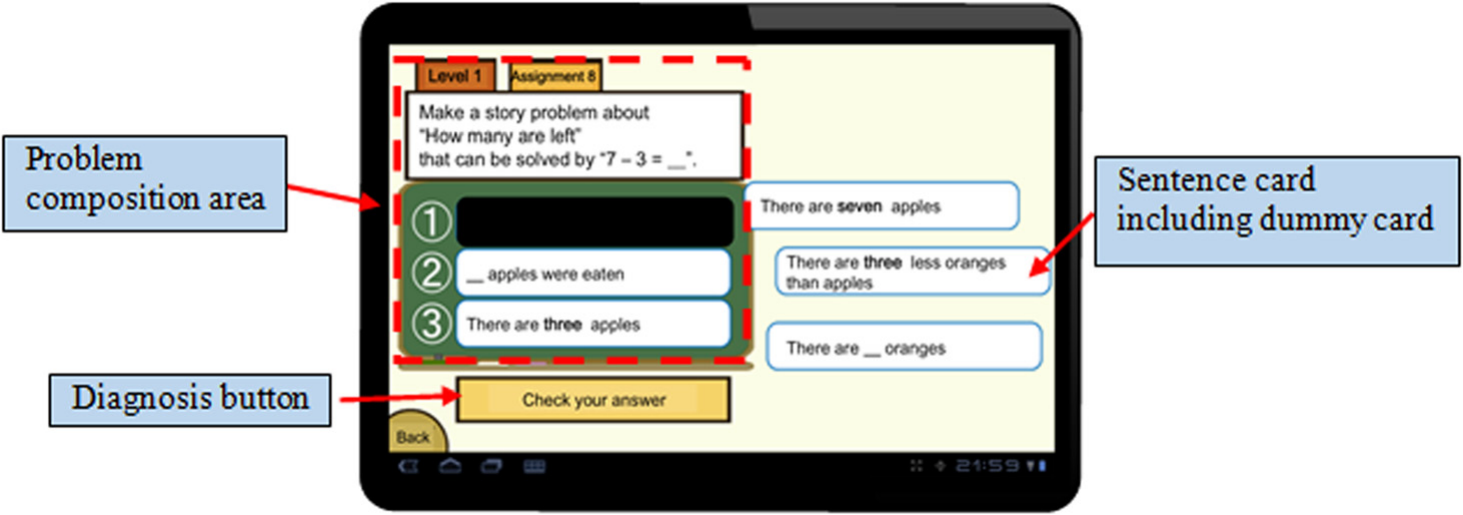
The interface of MONSAKUN

The MONSAKUN interface consists of three components: the problem composition area, the sentence cards, and the diagnosis button, as shown in Fig. 1. MONSAKUN gives learners problem-posing exercises, in which they pose a problem that satisfies certain requirements. The problem composition area on the left side of the interface consists of the requirement part and the card slot part to pose a problem. The requirement part includes two types of requirements: (1) a story type that the required problem to be posed must belong to and (2) a numerical formula that must represent the numerical relation in the required problem. Learners try to formulate the required problem to put sentence cards in the card slot at the bottom of this area. Sentence cards are presented on the right side of the interface. Learners can move the cards by dragging and dropping them freely to the card slots. There are more than three cards provided to the learners, which means that not all the cards included are necessary to complete the required problem. We call such cards “dummy cards,” and they are intentionally included to test learners and check for their understanding. For example, an overlooking of the story type or confusion about the formula may lead to problems completing the exercise correctly.

The last component is a button located under the problem composition area called the diagnosis button. The diagnosis button is used to check the answer of the combination of sentence cards chosen by the learner. The learner selects several sentence cards and arranges them to pose a problem in the correct order. Putting a sentence card into a card slot and removing a sentence card from a card slot are the basic actions of learners on MONSAKUN. MONSAKUN records the results of learners’ problem-posing activity, which are combinations of cards arranged in the card slots.

### The triplet structure model

MONSAKUN has been developed as an interactive environment for learning by problem-posing with sentence integration based on the triplet structure model (Hirashima et al. 2014). This model is an arithmetical word problem model that is solved by one arithmetical operation composed of three quantities: operand, operand, and result quantity. In this model, all word problems are composed of two “existence sentences” and one “relational sentence.” Although an existence sentence can be used in any story type, a relational sentence is used only in one specific story type. There are four story types: combination stories, increase stories, decrease stories, and comparison stories.

Combination and comparison stories are composed of two existence sentences and one relational sentence. One existence sentence describes the quantity of an object, and the other existence sentence describes the quantity of another object. The relational sentence of combination and comparison stories expresses the total quantity and difference of the two objects, respectively. The following example is a typical arithmetical problem that is expressed by the triplet structure model in a combination story:

{There are 3 white rabbits (first existence sentence). There are 5 black rabbits (second existence sentence). There are 8 white and black rabbits altogether (relational sentence).}

In this story, the changes to the order of the sentences do not affect the problem story. For example, when the sentence “There are 8 white and black rabbits altogether,” is placed at the beginning, followed by “There are 3 white rabbits,” and ends with “There are 5 black rabbits,” this new composition still forms the problem story.

Increase and decrease stories are also composed of two existence sentences and one relational sentence. One existence sentence describes the quantity before an increase or decrease, and the other existence sentence describes the quantity after an increase or decrease. Each existence sentence only describes the quantity of an object. The relational sentence describes the quantity of the increase or decrease. The relational sentence expresses the relation between before and after the quantity of the increase or decrease. The following is a typical arithmetical problem that is expressed by the triplet structure model in a decrease story:

{There are 5 apples (first existence sentence). 2 apples are eaten (relational sentence). Now there are 3 apples (second existence sentence).}

In this case, any change in the sentence order will affect the problem story. For example, if the relational sentence “2 apples are eaten” is placed at the beginning, this composition cannot form the problem story. It is wrong in the story when suddenly two apples are eaten without explaining their prior existence. The initial quantity of apples is required. Therefore, when learners try to pose the sentences in the wrong order in the increase and decrease story types, they cannot understand the story.

**Fig. 2:**
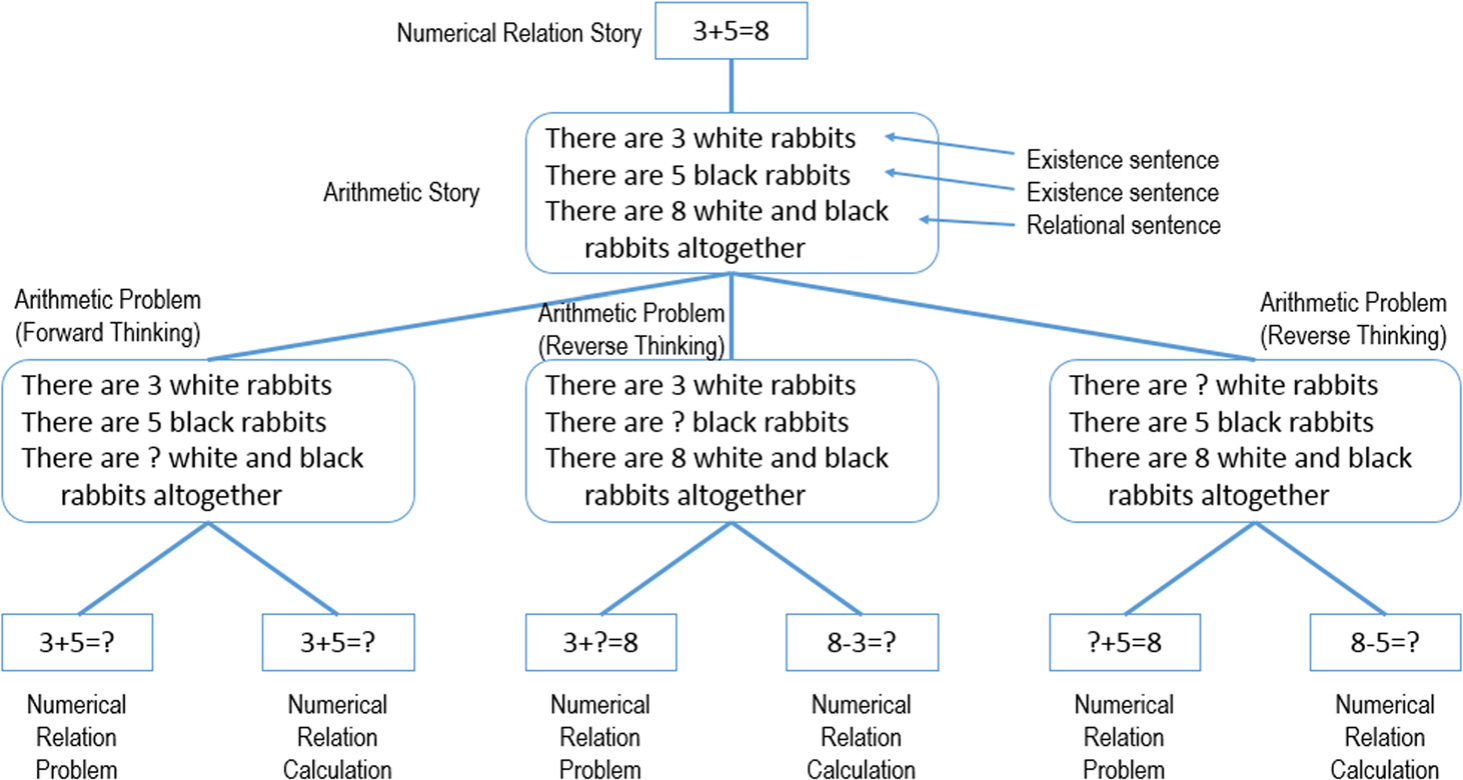
Relation between arithmetic story and problem

In this model, depending on the combination of two existence sentences and one relational sentence, the role of each sentence is changed. The relation between an arithmetic story and other problems is shown in Fig. 2. Based on the answer to the arithmetical word problem, it is possible to make a numerical relation and a cover story composed of all known numbers. We call this cover story an “arithmetic story,” and this numerical relation a “numerical relation story.” Then, the numerical relation in the problem including the unknown number is called a “numerical relation problem,” and a numerical relation used in the calculation is called a “numerical relation calculation.”

When the numerical relation problem is the same as the numerical relation calculation, understanding the cover story is the same as solving the problem. We call such a problem a “forward-thinking problem.” However, when the numerical relation problem is different from the numerical relation calculation, it is necessary to transform the numerical relation problem to a numerical relation calculation after understanding the cover story. We call such problem a “reverse-thinking problem.” Because learners are required to comprehend the relation between two structures, reverse-thinking problems are more difficult than forward-thinking problems.

### The constraints based on the task model

The task model of problem-posing via sentence integration was developed based on the consideration of problem types in the triplet structure model (Hirashima and Kurayama 2011). Based on the task model, we have devised five main constraints to be satisfied by each posed problem, which are (1) calculation, (2) story type, (3) number, (4) objects, and (5) sentence structure. When all five constraints are satisfied, the learner has succeeded in posing a correct problem according to the assignment requirements. When less than five constraints are satisfied, it shows that the learner has acquired a level of understanding in the structure of the arithmetic word problem; however, the final problem does not yet satisfy the requirements. If there are no constraints satisfied by the learner, it shows that the learner is unable to understand the structure of the arithmetic word problem.

Examples of compositions of sentence cards and their satisfaction of constraints are presented in Table 1. The requirement of this assignment is: Make a word problem about “How many are there in all” that can be solved by “8–3”. This assignment is a combination story. The first example only satisfies one constraint, number. In this case, the learner only focuses on the number. There is no story that can be built from this composition, nor the calculation and sentence structure. It can be calculated and well structured when it consists of two existence sentences and one relational sentence, instead of just existence sentences. In addition, there is no relation between objects in the card’s composition. They are independent objects that consist of white, black, and brown rabbits. The second example satisfies two constraints: object and sentence structure. There is a relationship between objects (white and black rabbits), and the structure of the sentence cards consists of two existence sentences and one relational sentence. However, the calculation, the story type, and the number are not fulfilled. There is no number “8,” as it causes a calculation process that cannot be done and the number constraint is not fulfilled. Concerning the story type, this is a comparison story, instead of a combination story. The third example is an example of a state that satisfies all constraints.

**Table 1:** Examples of compositions of sentence cards and their satisfaction of constraints

No	Card’s composition	Constraint				
		Calculation	Story type	Number	Object	Sentence structure
1	There are 8 white rabbits			x		
	There are 3 brown rabbits					
	There are _ black rabbits					
2	There are 3 white rabbits				x	x
	There are 3 more white rabbits than black rabbits					
	There are _ black rabbits					
3	There are 3 white rabbits	x	x	x	x	x
	There are _ black rabbits					
	There are 8 white and black rabbits altogether					

### Visualizations of the problem-posing process

In order to achieve our research goal, we first collected log data of learners’ activity from MONSAKUN. Then, we designed a formulation to encode the problem-posing process. At this stage, a transformation from a sentence card to the number representation was applied. After that, we traced the problem-posing activity sequences recorded in the database. In the next stage, we calculated two kinds of values: support and distance values. These two values were used to measure the frequency of learners who attempted an action and to measure the distance from an action to the correct answer, respectively. Then, we visualized the values as graph representations. Finally, we analyzed our findings based on the visualizations.

### Log data in MONSAKUN

**Fig. 3:**
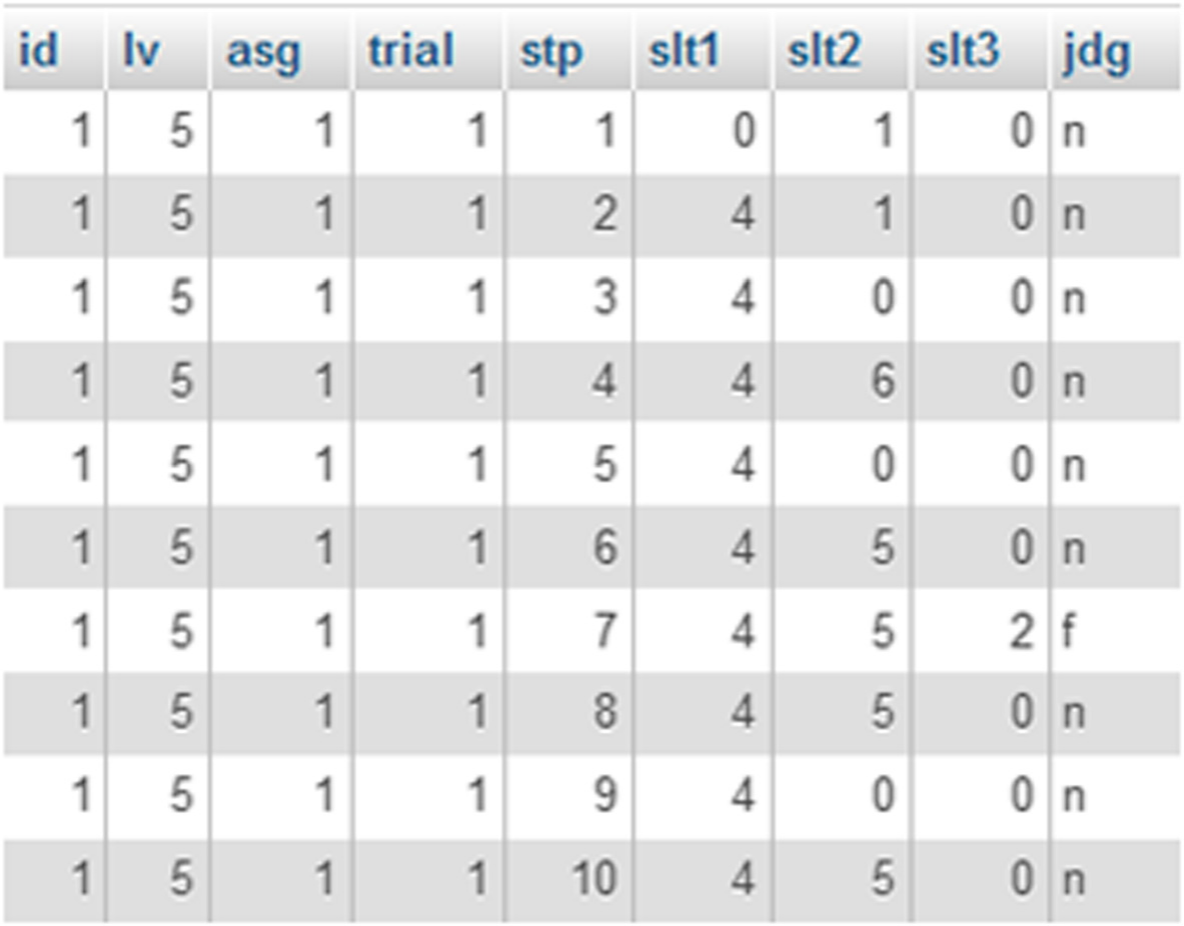
Example of log data from learners actions

Each learner’s actions on MONSAKUN were logged into a database. The raw data was coded as a series of events, where the events were encoded as id, lv, asg, trial, stp, slt1, slt2, slt3, and jdg. The code “id” showed the learner ID. The code “lv” is the difficulty of the problem-posing task and “asg” is the number of the assignment. The code “trial” is learners’ trial, and “stp” shows the sequence number of the actions. The codes “slt1”, “slt2,” and “slt3” denote the location of sentence cards of the first place, second place, and third place, respectively. The last code, “jdg,” shows the type of action, for example, uncompleted slot action, failed action, or successful action. We present a sample of log data from learners’ actions in Fig. 3.

### Formulation of the problem-posing process

MONSAKUN records learners’ problem-posing activities as combinations of cards set in the card slots. The product of an activity is a combination of cards, which is called the “state” in the problem-posing process by learners, as shown in Table 2. Type 1 is the uncompleted state, which is composed of less than three cards. This type includes, at least, one empty card slot. The empty card slots are represented by zero (the state shown in brown). Type 2 is the completed state, which is composed of three cards. However, the learner did not ask the system to diagnose the posed problem. This type is coded by a combination of three card numbers and followed by the string “[u]” (the state shown in black). Type 3 is the failed state. Although this is also composed of three cards, the system diagnosed it as a failure. This type is coded by a combination of three card numbers and followed by the string “[f]” (the state shown in red). The last one is the successful state (type 4), which the system diagnosed as the correct answer. This type is coded by a combination of three card numbers without being followed by any other string code.

**Table 2:** Example of each state type

Type	Definition	Example	Description
1	Uncompleted state		Slot 1 is empty, slot 2 is occupied by card 1, slot 3 is empty
2	Completed state without push button		Slot 1 is occupied by card 4, slot 2 is occupied by card 1, slot 3 is occupied by card 3, and without checking the answer
3	Completed state and gets wrong answer		Slot 1 is occupied by card 3, slot 2 is occupied by card 1, slot 3 is occupied by card 5, and check the answer then gets wrong answer
4	Completed state and gets correct answer		Slot 1 is occupied by card 3, slot 2 is occupied by card 1, slot 3 is occupied by card 2, and check the answer then gets success

Based on the model, all possible combinations of cards and transitions among them could be clearly defined as a network of states. We call this network the “problem states space.” All the actions of learners could be mapped to a transition from one state to another in this network. All possible states can be obtained by combining all the available sentence cards, including the empty slot. Each state represents a basic unit of thinking, and a problem state space provides the range of thinking in a problem-posing assignment.

The possible combination starts from state 000, the initial state, which means that all slots are empty (root state). It continues with combinations of one card slot filled, two card slots filled, and three card slots filled (complete state). There is a constraint that must be satisfied to generate all possible states. Each card can be used only once. For example, it is impossible to create the state 131, which means that the first card is used twice, in the first slot and the third slot. However, it becomes possible to make a combination of more than one empty slot, for example, 001, 002, 003, and so on.

In the next step, we connected each state in accordance with the proper conditions. The proper condition is one where there is a relation between the situation before and after an action. For example, we connected a situation where all slots are empty with a situation where one card slot is filled. It was impossible to connect a situation where all slots are empty with a situation in which two slots are filled with cards because there is one situation that elapsed. As a concrete example, we could not connect state 000 to state 012 because there is one step that elapsed before the state 012. The state that may be done before state 012 is state 001 or state 002.

**Fig. 4:**
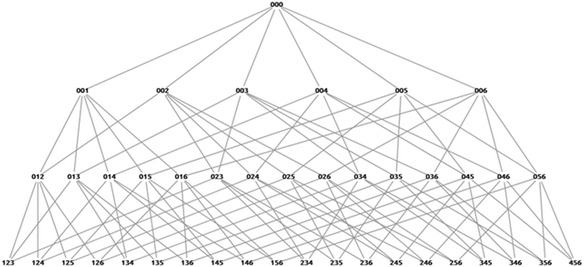
The graph of states space for combination story type with 6 available sentence cards

The example of all possible states from six available cards with a combination story is shown in Fig. 4. Since there is no required order of cards in the state, states that have the same composition are combined into one state. For instance, states 013, 031, 103, 130, 301, and 310 are combined into state 013. As a result of combining states, we get 42 states in total for combination and comparison stories. While for the increase and decrease story types, we get 136 states in total from 5 available sentence cards.

### Tracing the way of thinking: the sequence of states

**Fig. 5:**
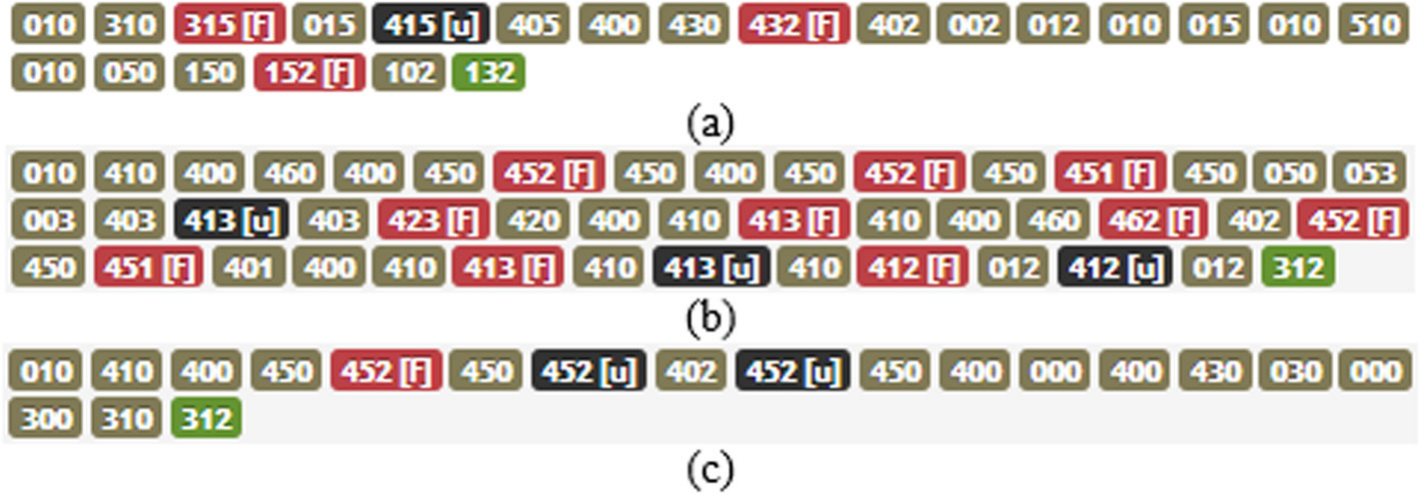
The examples of sequence of states (**a**) The sequence of learner 1 (**b**) The sequence of learner 2 (**c**) The sequence of learner 3

In order to complete an assignment, the learners tried varying compositions of the cards, in order to generate a particular state according to what they thought. They continued to change the composition of the cards until they reached the correct card composition. Every state that occurred for learners was stored by the system. Thus, we had an order of each state called a “sequence of states.” A sequence of states is a collection of states that are sorted based on the sequence of learners’ activity. This sequence reflects the learners’ thinking processes. Examples of sequences are shown in Fig. 5.

**Fig. 6:**
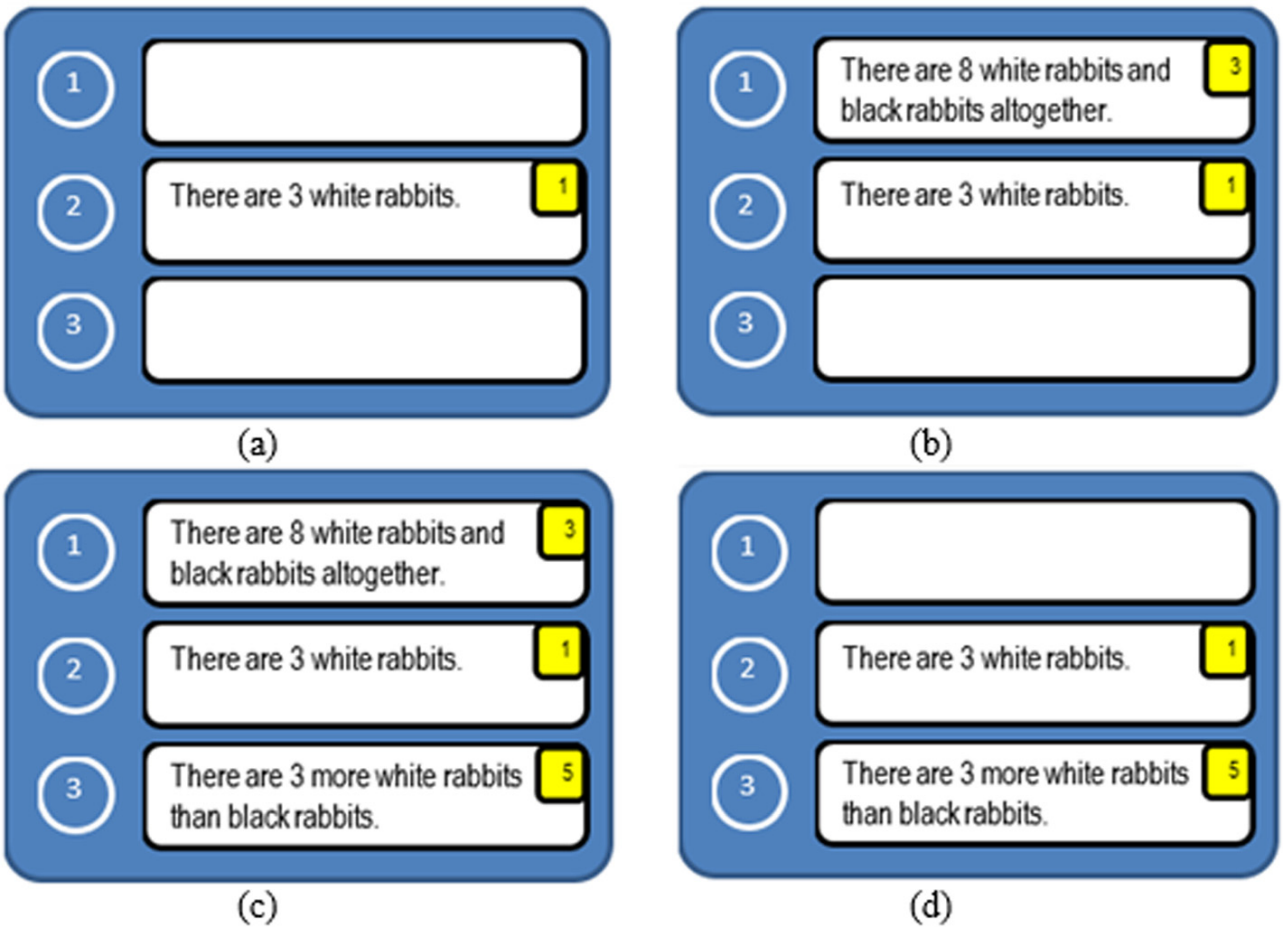
Part of a sequence (**a**) State 010 (**b**) State 310 (**c**) State 315 (**d**) State 015

**Fig. 7:**
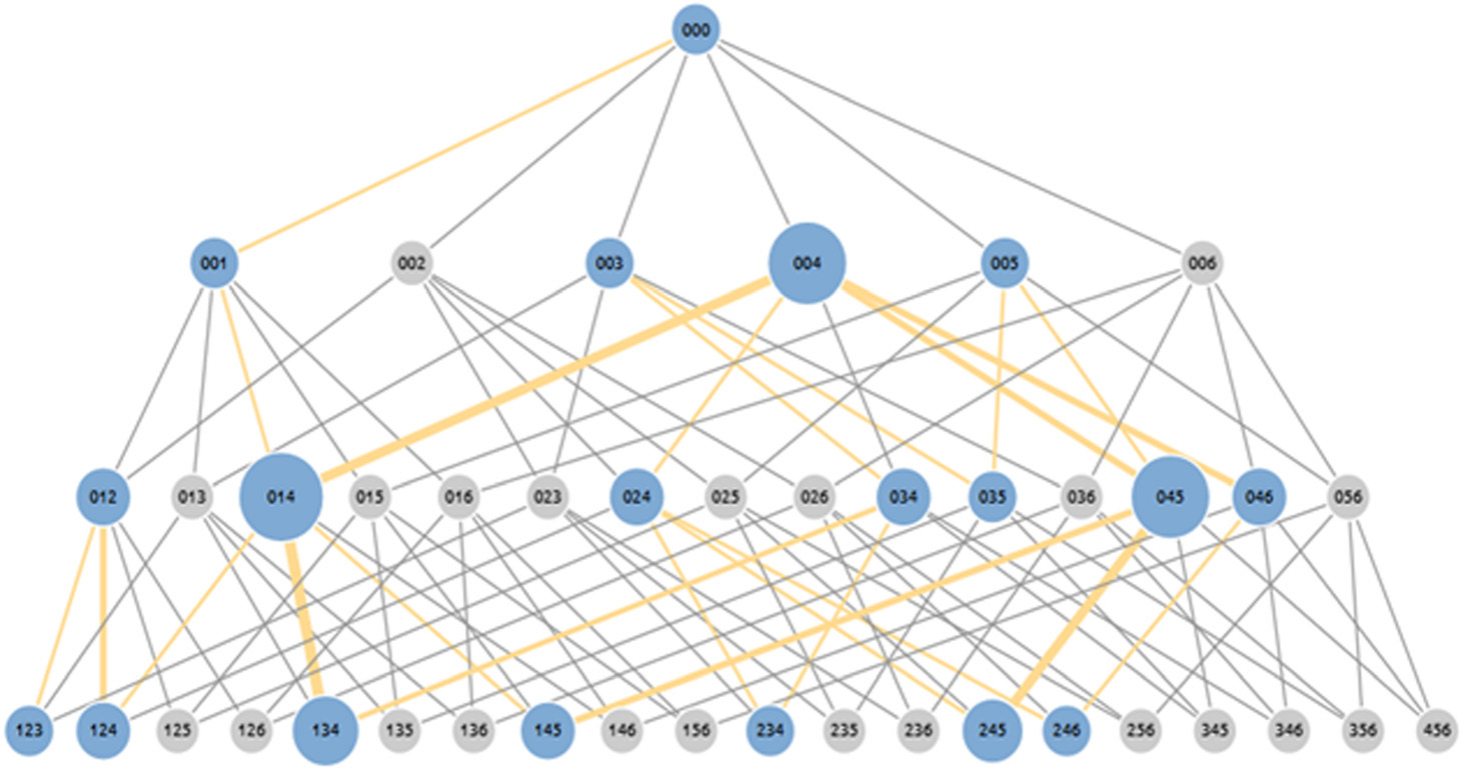
An example of mapped a sequence to the problem state space

There are three examples of sequences that have a different number of states, as shown in Fig. 5. The first sequence had 22 states, as shown in Fig. 5a; this means that the sequence comprises 22 steps to reach the correct answer. The second and third sequences had 45 steps, as shown in Fig. 5b, and 19 steps, as shown in Fig. 5c, respectively. Four example steps in the first sequence are shown in Fig. 6. The first sequence begins with the state 010, as shown in Fig. 6a; this means that the learner put the first card in the second slot. The next state is the state 310, as shown in Fig. 6b. In this state, the learner put the third card into the first slot. Next, the learner put the fifth card into the third slot, followed by pressing the diagnosis button, at which point the learner achieves the wrong answer. Representation of this state is 315(f), as shown in Fig. 6c. The learner tried to correct the error by taking the third card from the first slot; this condition turns the state into 015. The condition of the state 015 is shown in Fig. 6d. The complete steps of the first sequence, as shown in Fig. 5a, could be mapped to the problem state space, as shown in Fig. 7. The blue nodes show visited states. The yellow links show the relations between the visited states, and the thickness represents how many steps the learner followed to get there. On the other hand, the gray nodes show the states that were never arranged by the learners. Blue nodes and yellow links represent what the learner considered before he/she arrived at the correct answer.

### Support value and distance value

We propose two kinds of values to be visualized: support value and distance value. In this study, support value denotes the number of learners who attempted to pose a state. This value aims to show how many learners arranged the state. On the other hand, distance value denotes the average number of steps it took for learners to reach a correct answer from a state. This value aims to show how far or close the learner came to the correct answer; thus, it is called the distance of state.

The sequences of states as shown in Fig. 5 are sequences that have been carried out by different learners. State 010 shown in Fig. 5a occurs four times to achieve the correct answer, they are at the 1st step, the 13th step, the 15th step, and the 17th step that causes distances of 21, 9, 7, and 5, respectively. We obtained the distance value of state 010 in this sequence by calculating the average of all distance values. State 010 in this sequence has a distance value of 11.

### Graph visualization

A sequence has several states as objects that are linked by ordered steps. The first step linked to the second step, the second step linked to the third one, and so on. For this reason, we proposed the graph visualization, which shows the states and their relations. Moreover, information visualization is best represented in graph structures, which act as bridges between the visualization and the graph drawing field (Gröller 2002). Rabbany et al. (2011) used graph representation to visualize overall snapshots of the students’ participation in the discussion forums and give the instructor a quick view of what is under discussion in online courses. In this study, such as in Johnson et al. (2011), we designed a graph where each node represents a state, and each link represents an action that takes a learner from one state to the next one. The graph gives an overview visualization of all relations between the previous state and the next state in a sequence.

### Trap state: a finding of characteristic states in problem-posing

A state performed by learners is the result of their thinking. When learners choose to put one card into an empty slot due to its possible potential, it has a consequence. Similarly, when learners tried to take out a card that has been installed in one slot, it will lead to consequences too. The consequences could move learners away from the correct answer. In this case, the learners are stuck in a condition where they would have to do more steps to reach the correct answer. In other words, the learners are trapped in the state that distanced them from the correct answer. Many learners found themselves in this state.

We defined two important circumstances related to learners who were stuck in these conditions. The first circumstance is related to learners who were in a condition where there was no dummy card contained in the state. The second circumstance is related to learners who were in the condition in which there was at least one dummy card found in the state. Thus, we defined a state with no dummy card that could lead learners to doing many steps to get to the correct answer and reached by many learners as “confusing state,” while a state contained at least one dummy card where it could lead learners do many steps to get to the correct answer and reached by many learners as an “actual trap state.”

### Application of the visualizations to a case study on the practical use of MONSAKUN

#### Participants and materials

The current study collected the data of 39 participants to study learners’ activity on MONSAKUN. The participants were Japanese students in the first grade of elementary school; their average age was 6 years old. Learners had already studied problem structure on the blackboard using several sentence cards that were parts of problems (Yamamoto et al. 2012). These cards were provided to the learners as a request of problem-posing. In order to promote deeper learning, MONSAKUN was used as an interactive learning environment to exercise and receive lessons on problem structure as part of their regular classes. Each learner was asked to create a story problem using the sentence cards based on calculation expressions. They had to select a sentence card, move it to the available slot, and complete all three slots. There were five or six cards provided to them by the system. When learners finished posing the problem, they pushed the diagnosis button under the problem composition area. Then, the system diagnosed the combination of the sentences, showed the results, and sent a message to help with the learner’s problem-posing in another window.

Through practical use of MONSAKUN, it was confirmed that the first-grade students were able to successfully pose problems in this learning environment (Yamamoto et al. 2012). Additionally, the preliminary research concerning learners’ answers and the completed posed problems was conducted by Hasanah et al. (2015b). They found that learners did not pose problems randomly, but did so with some understanding. In this study, by using the same data, deep analysis of learners’ thinking was proposed. We would like to study not only the completed posed problems but also the uncompleted posed problems. We visualized the learners’ activity, including every action, in order to extract some information from it. We believe that the visualization and analysis could be used to support learners due to a bottleneck in thinking.

We (including the homeroom teacher of the elementary school where MONSAKUN was used) designed a teaching method based on the problem structure expressed in the task model of problem-posing (Hirashima and Kurayama 2011), and MONSAKUN was developed based on it. We used the latest version of MONSAKUN, called MONSAKUN Touch (Yamamoto et al. 2012), in order to allow the learners to use MONSAKUN not only for the exercises but also for lessons on problem structure as part of their regular classes.

In practical use, learners used the MONSAKUN as an introduction to problem-posing (5–10 min) at the beginning of class. Then, the teacher taught the problem structure on the blackboard by using several sentence cards that were parts of problems (20–35 min). The teacher prepared the requirement card and several sentence cards. These cards were provided to the learners as a requirement of problem-posing. First, the teacher presented one sentence card to the learners from the prepared sentence cards, and the teacher explained the elements of the sentence card. The sentence card was composed of object(s), a value of the object(s), and the predicate. Second, the teacher presented another sentence card from the prepared sentence cards. Then, learners gave responses about whether the presented sentence card was necessary to pose the problem or not, and they also expressed the reason why the card was necessary. Finally, the teacher explained the problem structure based on their answer. Through this teaching, the learners understood the following things: (1) a problem was composed of two existence sentences and one relational sentence; (2) a sentence represented a story type and a relation among sentences, and there was a required sentence order based on the specific story type; and (3) a numerical formula was needed to represent the story type and find the answer. In that time, learners actively engaged in the lesson to express their opinions and ideas about posing the arithmetical word problems provided on the blackboard. Finally, at the end of class, learners used MONSAKUN to practice problem-posing exercises (5–10 min). When a learner finished all of the 12 assignments in the level, they could complete the challenge for that level. As a result, some learners in this time allocation were able to attempt the same problem a maximum of three times. Therefore, we collected the data from three trials of the same problem by the same learners.

Levels one to five were the same in terms of posing problems from a card set, but they had different requirements. Levels one to four provided the numerical formula of the story, while level five was required to consider the unknown number. Hasanah et al. (2015b) reported that the average steps and mistakes in level five were significantly different from the others. In this study, we present an investigation of the first assignment in level five, which contained a combination story problem. The requirement of this assignment was make a word problem about “How many are there in all” that can be solved by “8–3.” There were six sentence cards that could be used by learners. The sentences for each card, from the first card to the sixth card, were the following:

- 1. There are 3 white rabbits.
- 2. There are _ black rabbits.
- 3. There are 8 white and black rabbits altogether.
- 4. There are 8 white rabbits.
- 5. There are 3 more white rabbits than black rabbits.
- 6. There are 3 brown rabbits.

In this assignment, the correct answer consisted of card 1, card 2, and card 3 (the sentences appearing in bold).

### Support graph and distance graph

**Fig. 8:**
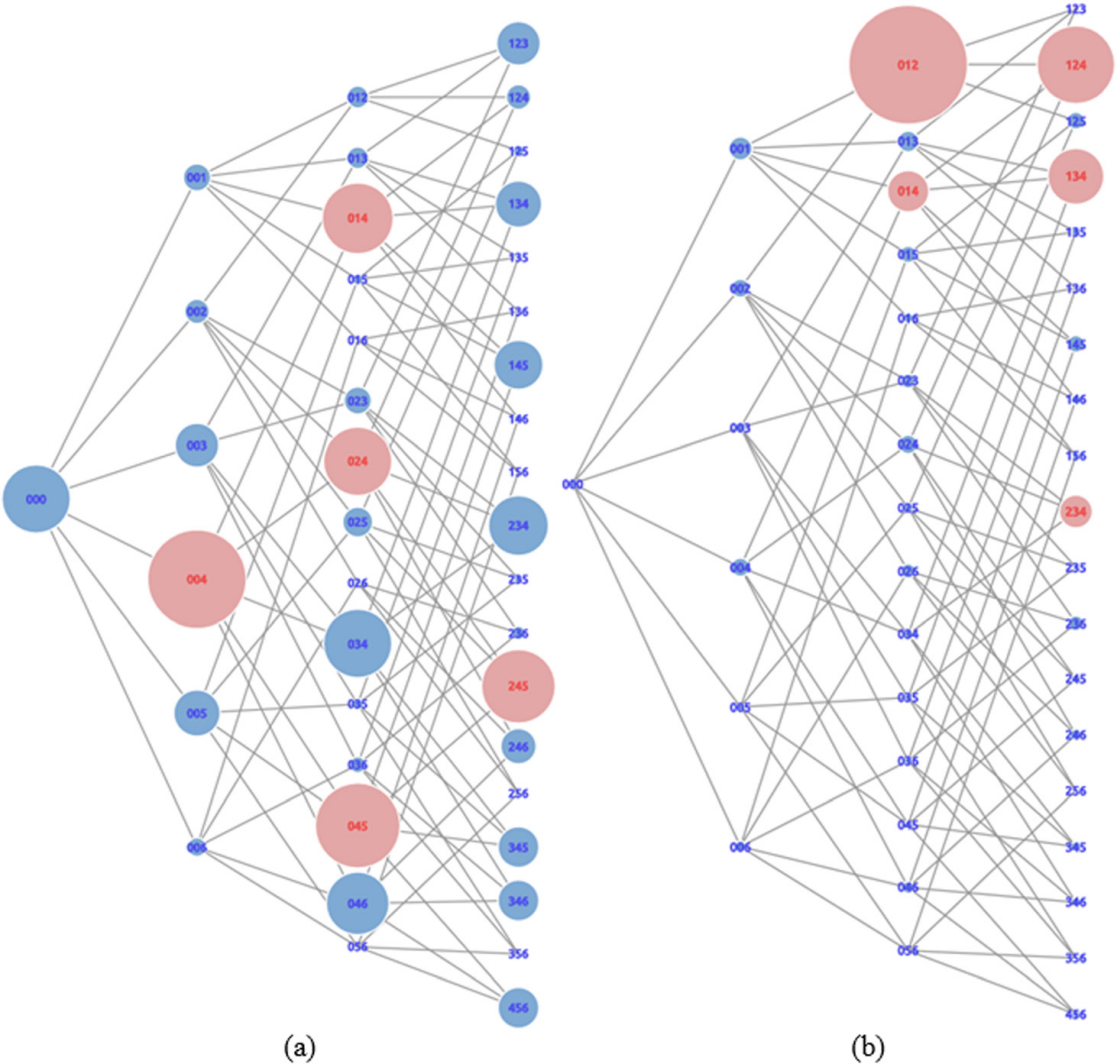
Support graph and distance graph of first trial (**a**) The support graph (**b**) The distance graph

In this section, we describe two kinds of graphs, “support graphs” and “distance graphs,” based on their values. For the first trial, Fig. 8a, b shows examples of support graphs and distance graphs, respectively. A support graph displays the support value of the states performed by learners. This graph aimed to visualize states that have the number of supports shown by the size of the node. The node with a larger size has a higher number of supports than the node with smaller sizes. A distance graph displays the proximity of states to the correct answer. This graph aimed to visualize the average number of steps of a state to the correct answer indicated by the size of the node. The larger the node size, the greater the number of average steps. It means that a large-sized node had a longer distance to the correct answer on average.

The value of each state in both types of graphs was normalized by scaling 0 to 1. We discarded the node that had a value of zero, which means the learners had never done that state. We focused on the state that had already been made by learners. We also implemented two different colors for nodes. The color was determined based on the top five support and distance values. The top five nodes were colored red, and the rest were blue. We did this on the grounds that the top five nodes are (1) states that had a high-value of support, as shown in Fig. 8a, and (2) states that had long distances from the correct answer, as shown in Fig. 8b. For that reason, we would like to focus on the red states for further analysis.

We argue that using these two graphs, based on the red states for both sides, valuable information could be discovered. For instance, state 004 is one of the principal states from the support graph. However, it does not have a high-distance value. This means that many learners made the state but they easily became aware of its incorrectness. On the other hand, state 012 is one of the principal states from the distance graph. However, it does not have a high-support value. It means that very few learners were able make it and felt that it was difficult. We need to find states where many learners feel challenged. For this purpose, we combined both graphs.

Based on the value of its support, the red states on the support graph shown in Fig. 8a are 004, 045, 245, 014, and 024, which have support values of 37, 32, 28, 27, and 26 respectively. This means that state 004 was attempted by 37 learners, state 045 was attempted by 32 learners, etc. On the other hand, the red states on the distance graph shown in Fig. 8b are 012, 124, 134, 014, and 234, which had the average distance to the correct answers of 68, 44, 32, 24, and 19, respectively. This means that when learners were in state 012, they took 68 steps to reach the correct answer on average. Similarly, state 124 required 44 steps, state 134 required 32 steps, etc. Highlighted states on the distance graph are strong candidates for trap states because learners got stuck and had to do many steps to reach the correct answer. However, this was not enough to identify that a state was a trap state. As defined previously, that trap state is a state that not only leads learners to do many steps but also requires learners to be supported by others. Therefore, by combining the distance graph with the support graph, the actual trap states were revealed.

The most distinct state in the distance graph is state 012 (the largest node shown in Fig. 8b). This situation shows that the state 012 could potentially be a trap state for many learners. However, when we looked at the support value, this state was only supported by a few learners (shown by the little blue node in Fig. 8a). Although state 012 was one that required many steps to reach the correct answer in this data, there were not many learners who could arrange this state. The same thing occurred in state 124, 134, and state 234. The rest state with the red color shown in Fig. 8b is state 014. When learners were in this state, they were required to complete 24 steps to reach the correct answer on average. Moreover, this state was also reached by many learners, as shown in red in Fig. 8a. This means that, for many learners, they tended to do more steps and move further away from the correct answer when they were in state 014. This state was supported by 27 learners. Besides, state 014 contained a dummy card. Thus, this state could be said to be an actual trap state. In other words, by using visualization, it was revealed that the red colored state shown in the Support and Distance Graph was an actual trap state.

### Trap graph

**Fig. 9:**
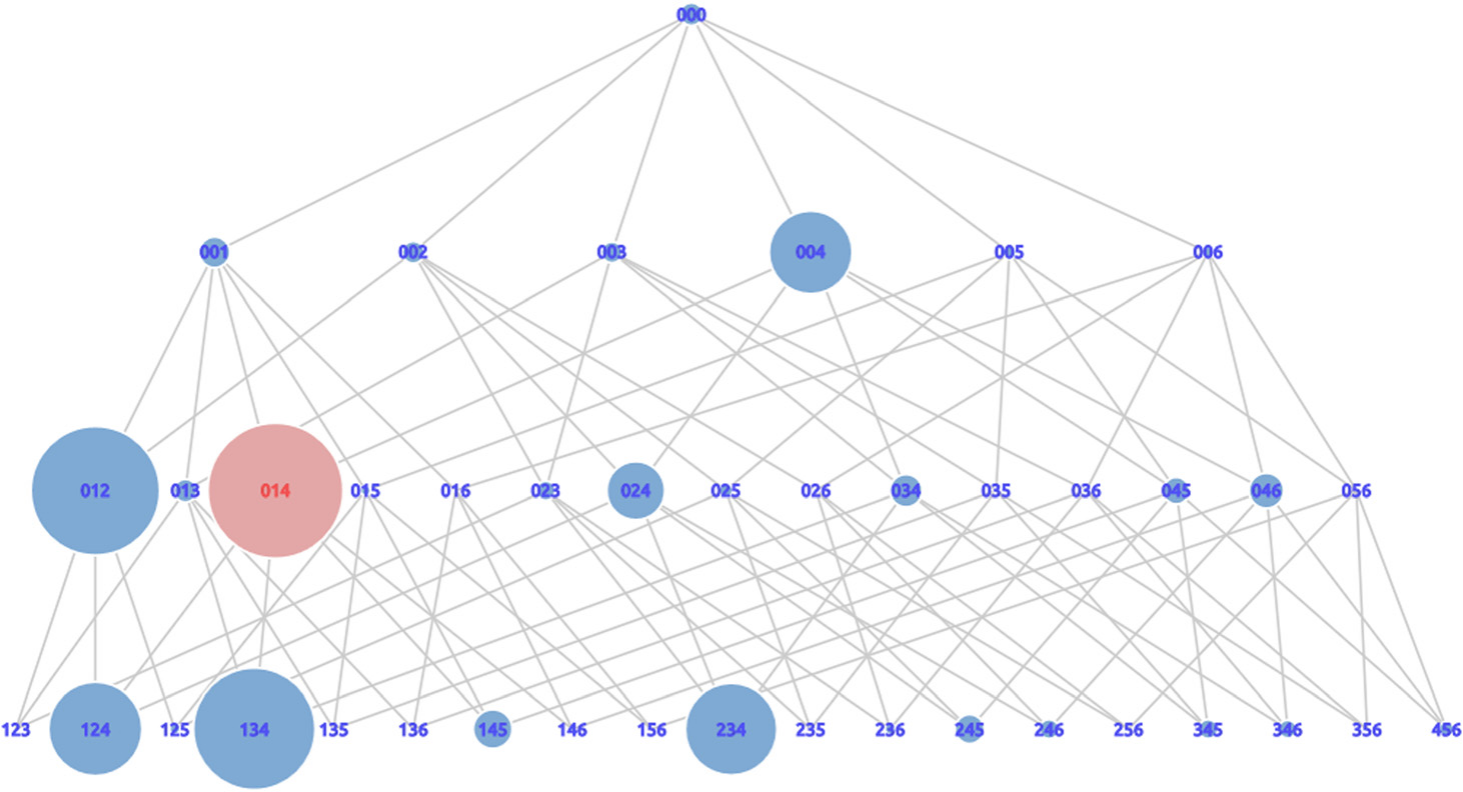
Trap graph of the first trial

In this section, we present another visualization called the “trap graph.” It was obtained by multiplying the support value and the average distance value together. We refer to this multiplication result as the trap value. We also normalized the trap value by scaling 0 to 1. The trap graph shown in Fig. 9 provides information about the actual trap state in the first trial. The actual trap state is displayed by a red node in the graph. From this graph, we also obtained the result that the actual trap state was state 014 as the highest trap value in the first trial. The result of the actual trap state in the trap graph showed the same result when we used the support and distance graph. We argue that the trap graph could be used to represent both the support graph and the distance graph. For this reason, we use the trap graph to detect the actual trap states in the second and third trials.

**Fig. 10:**
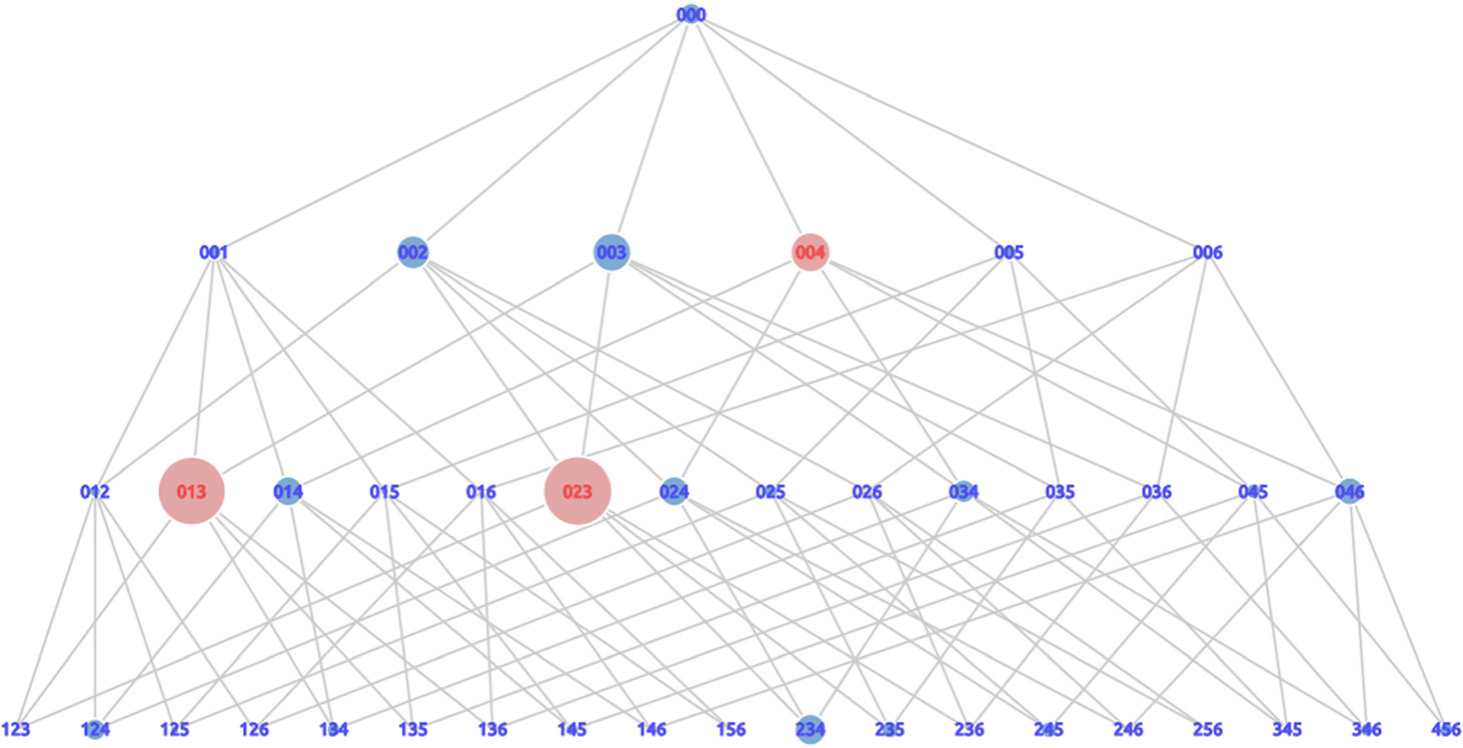
Trap graph of the second trial

In the second trial, we found that the two highest trap values were states 023 and 013 shown in Fig. 10, which did not contain any dummy cards. State 023 contained card 2 and card 3, both of which were correct cards. Also, state 013 contained card 1 and card 3, both of which were correct cards. Because these states did not contain any dummy cards, we refer to these states as confusing states. In these states, the learners are close to the answer. They have been on the correct path and only need one more step to reach the answer, meaning that learners need only one correct card to complete the problem. However, they did many steps to reach the correct answer. They needed 22 steps on average to get to the correct answer. However, there is nothing wrong with their actions to achieve the state 023 or state 013. Therefore, we argue that they were only confused, not trapped.

**Fig. 11:**
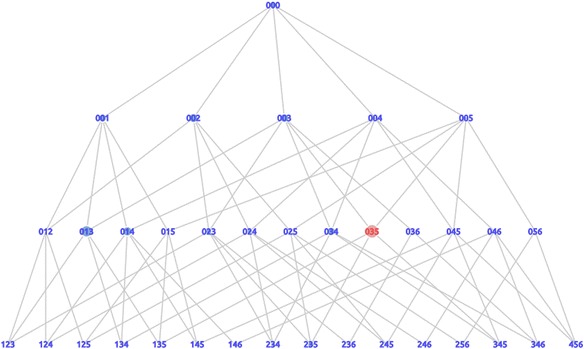
Trap graph of the third trial

The next high value is state 004, the red state shown in Fig. 10. This state was supported by many learners and required many steps to reach the correct answer. The difference with state 023 was that this state contained a dummy card. In this case, the learners chose the wrong card. The mistake of choosing this card caused the learners to be trapped in a condition that increased the distance to the correct answer. Thus, we detected state 004 as an actual trap state in the second trial of the assignment. The last trap graph generated from the third trial is shown in Fig. 11. State 035, the highest trap value on the graph, was detected as an actual trap-state.

## Discussion

The difficulty in this assignment was that learners were confused about the gap between the required story type of combination and the numerical expression of subtraction (8–3). Although subtraction implies the story type of decrease and comparison, in this case, learners must pose a problem of the combination story type. Before this assignment, learners had done assignments in which they could obtain the correct answers by arranging cards according to the order of numbers in the numerical expression. However, this was not valid for this assignment because the numerical expression did not express the story, but expressed the solution to evaluate the unknown number. Even if they made a strategy to arrange the cards according to the numerical expression from previous assignments, this strategy did not work in this assignment. Learners tended to make such a strategy (Hasanah et al., 2015a). To complete this assignment, for example, the learners needed to transform the numerical expression, “8–3,” into the numerical expression representing a combination story, such as “3 + ? = 8.” Moreover, learners could assign existence sentence cards to “3” and “?”.

The trap state in the first trial that tended to make learners do more steps and distanced them from the correct answer was state 014. State 014 consists of sentence card 1 (There are 3 white rabbits) and sentence card 4 (There are 8 white rabbits). This tendency demonstrated that learners tried to use the given numerical expression directly, “8–3,” and to assign cards 1 and 4 to “3” and “8,” respectively. Based on the available cards, it was reasonable that card 1 and card 4 had been chosen instead of card 2, which contained an unknown number (There are _ black rabbits), and card 6, which contained a different type of object from the others (There are 3 brown rabbits). In this situation, most of the learners became confused and stuck because they thought the correct answer was the number 8 on the numerical expression belongs to the existence sentence card (There are 8 white rabbits), but really they should have chosen the number 8 on the relational sentence card (There are 8 white and black rabbits altogether.) Thus, state 014 could also be explained as an actual trap state based on the triplet structure model. We thus confirm that, by using these visualizations, the actual trap state for learners could be detected.

**Fig. 12:**
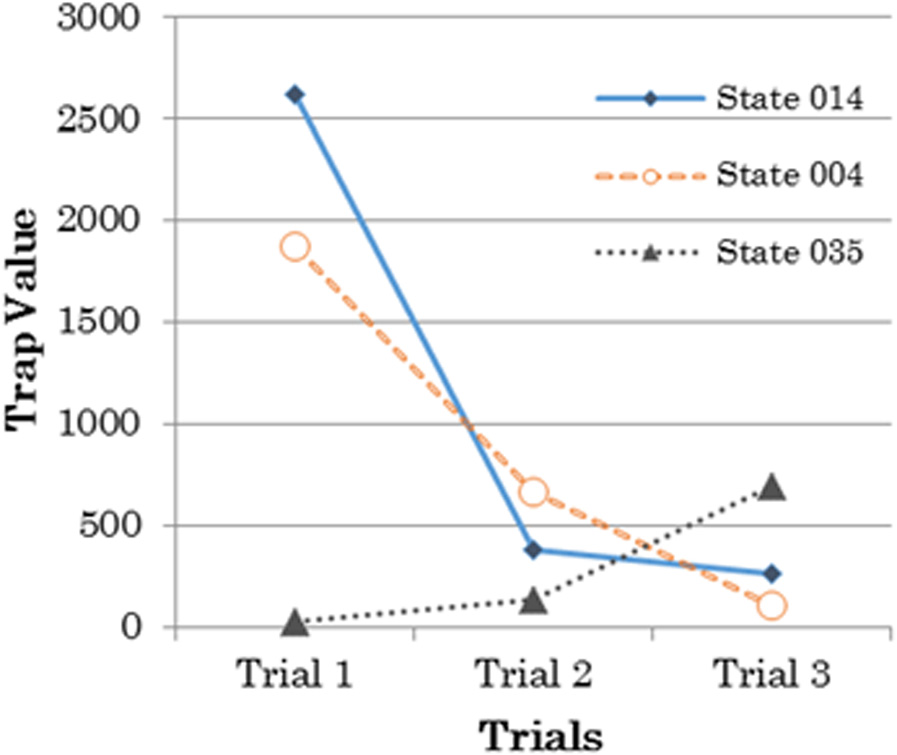
The trap value of trap state for each trial

Based on the trap graph in each trial, it was demonstrated that the principal trap state changed from the first trial to the second and third trials. In the first trial, the principal trap state was state 014. In the second trial and the third trial, the principal trap states were state 004 and state 035, respectively. More precisely, the changes of the actual trap states are indicated by the changes of the trap value for each trial, as shown in Fig. 12. The chart in Fig. 12 shows that state 014 had a trap value of 2621 in the first trial. This value is the highest value that enables state 014 to be detected as an actual trap state in the first trial. However, in the second trial, state 014 only has a trap value of 377. This value is lower than state 004, which has trap value of 662. Therefore, in the second trial, the actual trap state moved to state 004. In the third trial, the actual trap state changed again to state 035. This state has a trap value of 699, which increased from the previous trial where it only had a trap value of 136. On the other hand, the trap values of states 014 and 004 decreased to 266 and 101, respectively. We confirm that by using these visualizations, the actual trap state for learners in every trial could be detected. Moreover, the actual trap state changed from the first trial to the third trial, as summarized in Table 3. Table 3 also shows the number of learners who were in the state that was detected as an actual trap state. In general, it could be said that the number of learners in that state decreased.

**Table 3:** Actual trap states for each trial

Trial	Actual trap state	Number of learners
Trial 1	014	27
Trial 2	004	22
Trial 3	035	6

State 014 was found to be an actual trap state in the first trial. As discussed before, in this trial, learners used strategy to pose the problem by directly using the given numerical expression, “8–3.” The actual trap state in the second trial changed to state 004. State 004 only consisted of sentence card 4. (There are 8 white rabbits.) This was to encourage learners to try to change strategies to pose the problem and encourage them to move away from using numerical expressions. However, the strategy did not work because learners still kept trying to use card 4. There was still a decided tendency based on some sort of thinking (Hasanah et al. 2015b). They assumed that “8” in the numerical expression was considered to be an existence sentence. In this situation, most of them were confused and stuck because the correct answer with the number “8” was the number in the relational sentence. (There are 8 white and black rabbits altogether.) Thus, state 004 could also be explained as an actual trap state based on the triplet structure model. In the third trial, the actual trap-state changed and was detected as state 035. State 035 consisted of sentence card 3 (There are 8 white and black rabbits altogether.) and sentence card 5 (There are 3 more white rabbits than black rabbits.). This composition did not make a particular type of story. They put card 3, which reflected a combination story, and card 5, which reflected a comparison story, together in one state. Based on the number of learners shown in Table 3, we could say that a small number of learners still did not have a good understanding of the base structure of the problem.

Finally, we confirm that by using these visualizations, trap states for learners could be detected and they changed for each trial. This means that trap states in the previous trial did not remain trap states in the next trial. Moreover, the number of learners and actions decreased from one trial to the next. This indicates that the number of learners who had a good understanding of the problem structure increased, although there were small numbers of learners who were still confused.

## Conclusion

The current study presented visualizations that externalized the activity of learners in a problem-posing learning environment, where students posed problems based on the requirements of an assignment. The support graph provides the number of states that were visited by learners. The distance graph depicts the number of steps to the correct answer. These visualizations trace different aspects of the learners’ activity, and the combination of both visualizations produce the trap graph, which could detect the trap states for learners. Through this detection, we are able to receive information regarding situations in which many learners have some misunderstandings on the structure of the problems. The detected trap state is obtained based on the highest trap value. The current trap value is detected via tentative analysis, and thus, we need to sophisticate the calculation. At minimum, we need to distinguish between the high-supported state that was arranged by many learners and the low-supported state that was arranged by few.

In addition, we conducted three trials to investigate their progress of thinking in posing the same type of problem. From the analysis, we found that the trap state changed from the first trial to the third trial. Some learners had a tendency to change their strategy to pose the problem for each trial. The changes in their strategies caused the number of learners who were trapped to decrease from one trial to the next. We infer that the number of learners who had a good understanding of the problem structure increased as a result of this activity.

The ultimate goal of this line of research is placed in the context of exploring and mining the data in a problem-posing learning environment to obtain useful information to support learners. This research is still in its preliminary stages, and we believe that it still requires further analysis, such as evaluating these visualizations for all assignments. We also would like to explore ways to identify other significant actions. Furthermore, we are planning to use a data-mining method, such as sequential data-mining or the clustering method, to analyzing learners’ activities.
